# Rare case of aortic aneurysm with type A dissection (extending to right coronary artery) and severe AR in a nonhypertensive and non pregnant female

**DOI:** 10.1016/j.ijscr.2019.09.021

**Published:** 2019-09-25

**Authors:** Natraj Setty H.S., Rama Chikamuniswamy, Yeriswamy M.C., Rahul Patil, Veeresh Patil, Sathwik Raj, Santhosh Jadav, Balraju D., Krishna Murthy B.N., Babu Reddy, Srinivas B.C., Raghu T.R., Manjunath C.N.

**Affiliations:** Sri Jayadeva Institute of Cardiovascular Sciences and Research, Bangalore, Karnataka, India

**Keywords:** AAD, acute aortic dissection, AR, aortic regurgitation, ECG, electrocardiography, c-ANCA, anti-neutrophil cytoplasmic antibody, p-ANCA, perinuclear anti-neutrophil cytoplasmic antibodies, ANA, antinuclear antibody, 2D Echo, two-dimensional echocardiography, LV, left ventricle, MI, myocardial infarction, LMCA, left main coronary artery, RCA, right coronary artery, CT, computed tomography, CABG, coronary artery bypass graft, EF, ejection fraction, Aortic dissection, Aneurysm, BENTALL’S, CT aortogram

## Abstract

**Introduction:**

Typically a patient with acute aortic dissection presents with severe chest pain radiating to the back, tearing in nature. Rarely it can present as painless acute aortic dissection this is seen in iatrogenic cases or when associated with atherosclerosis, diabetes, or aortic aneurysm.

**Case presentation:**

We hereby present a case of a 32-year aged female who presented with dyspnoea & palpitations (NYHA III) from last 6 months, diagnosed to have Aortic Aneurysm with Type A dissection & Severe AR. She eventually underwent BENTALL’S procedure with CABG and had an uneventful recovery.

**Discussion:**

Clinical manifestation of Aortic dissection can be variable, therefore its diagnosis is challenging. 25% of cases, may have associated ECG changes suggestive of acute coronary syndrome leading to a possible misdiagnosis especially if associated ST elevation in ECG. Aorto arteritis is a non-atherosclerotic chronic inflammatory vascular disease of unknown etiology that affects the aorta, proximal parts of its major branches. In this case, there is a possibility that there was underlying spontaneous coronary artery dissection which in turn could be cause for silent ischemia in young women.

**Conclusion:**

Acute aortic dissection is a life-threatening disease with a high rate of cardiovascular morbidity and mortality. The most important and common risk factor is systemic hypertension which has been reported in the 70% of the patients with aortic dissection. Most of the aortic dissection observed in young women has been reported to be related to pregnancy. Dissection should be suspected during any acute coronary syndrome, particularly Inferior wall MI.

## Highlights

• Aortic dissection has a high mortality rate. It is common in elderly males. Aortic dilatation definitely increases risk but is not a must in every case. Clinical manifestation of Aortic dissection can be variable, therefore its diagnosis is challenging.
• Primary suspicion of Aortic dissection is usually based on a patient’s history which varies from typical complaints like a sharp, severe tearing kind of backache with chest pain to asymptomatic until associated with valvular regurgitation.
• Consequent treatment with antiplatelet, antithrombin and thrombolytic agents can cause life-threatening to bleed. Apart from good blood pressure control, prevention of aortic dissection can be done by elective aortic surgery in patients with dilated ascending aorta.
• The most important and common risk factor is the systemic hypertension which has been reported in the 70% of the patients with aortic dissection. Most of the aortic dissection observed in young women has been reported to be related to pregnancy.

## 1 Introduction

Aortic dissection has a high mortality rate. It is common in elderly males. Aortic dilatation definitely increases risk but is not a must in every case. Clinical manifestation of Aortic dissection can be variable, therefore its diagnosis is challenging. 25% of cases may have associated ECG changes suggestive of acute coronary syndrome leading to a possible misdiagnosis especially if associated ST elevation in ECG. In 1 to 5% cases there may be associated myocardial ischemia. Primary suspicion of Aortic dissection is usually based on a patient’s history which varies from typical complaints like a sharp, severe tearing kind of backache with chest pain to asymptomatic until associated with valvular regurgitation as in our patient. Spontaneous coronary artery dissection is a rare cause of myocardial infarction associated with significant high morbidity and mortality. It usually occurs in relatively young patients and it is frequently found at autopsy. This case has been reported in line with the SCARE criteria [1].

## 2 Case presentation

A 32-year-old female with no previous co-morbidities presented with dyspnea & palpitations (NYHA III) from the last 6 months. She was advised valve replacement surgery previously but she didn’t undergo. The patient presented now to our hospital in view of worsening dyspnea.

**Fig. 1:**
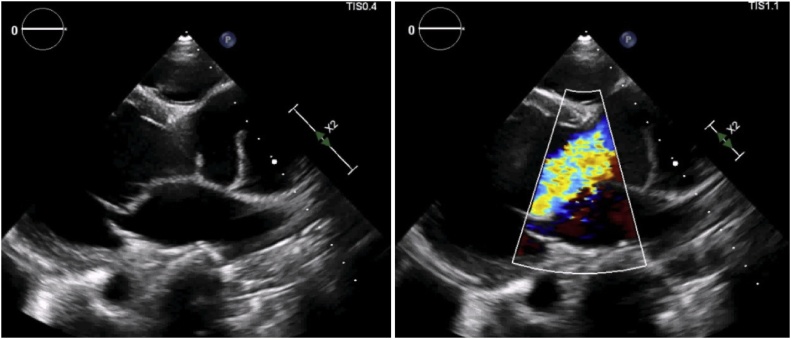
A: Parasternal long axis showing dilated aortic root & dilated LV. B: Color Doppler shows severe Aortic Regurgitation.

**Fig. 2:**
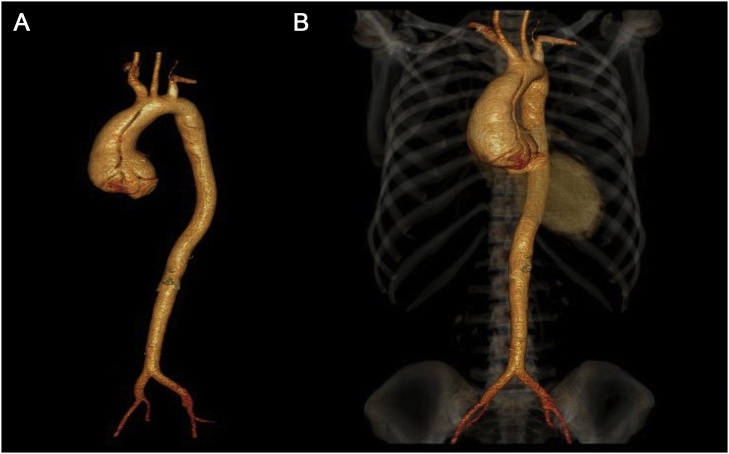
A and B: CT Aortogram shows dilated aortic root and ascending aorta which was 45 mm and 58 mm respectively and a dissection flap from just above the Left Main Coronary Artery (LMCA) ostium and involving the proximal segment of Right coronary artery (RCA) extending to the proximal aortic arch.

**Fig. 3:**
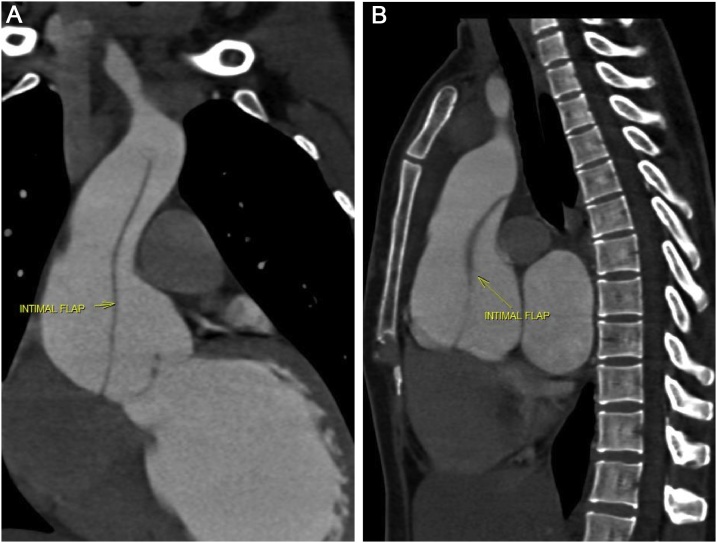
A and B: Coronal and Sagittal Multiplanar Reconstruction (MPR) of the 75% phase of the gated CT Aortogram study showing type A aortic dissection extending to the aortic root.

**Fig. 4:**
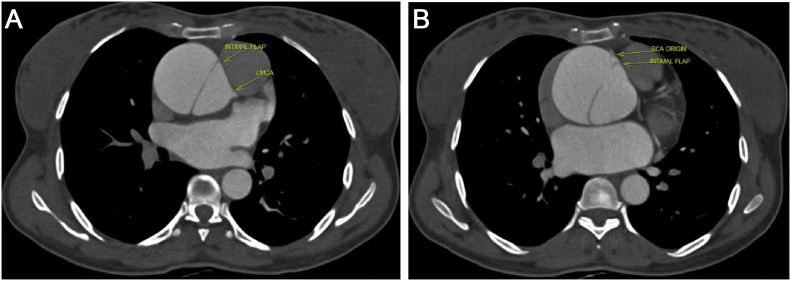
A and B: Axial Multiplanar Reconstruction (MPR) of the 45% phase of the gated CT Aortogram study showing the relationship of the intimal flap with the right coronary artery (RCA) and the left main coronary artery (LMCA).

On examination, pulse was 112 bpm, regular in rate and rhythm with increased force increased volume and normal tension, with collapsing character. Blood Pressure was 136/50 mmHg and Hill sign is positive with a difference of 60 mmHg. The patient was tachypneic at the time of examination. Auscultation revealed an early decrescendo diastolic murmur in the aortic area. Investigations including complete haemogram, renal function, liver function, electrolytes, serology for infectious diseases, connective tissue disease including c-ANCA, p-ANCA and ANA profile were normal. Chest X-Ray showed cardiomegaly with normal pulmonary vasculature. ECG showed sinus tachycardia with left ventricular (LV) strain with changes of evolved inferior wall MI. 2D echo showed dilatation of the aortic root and arch with severe AR, dilated LV with LV dysfunction (LVEF 40%), LVDd 6.2 cm^2^ and LVDs 5.1 cm^2^ (Fig. 1A & B) Hypokinesia of inferior segments. CT Aortogram revealed dilated aortic root (45 mm) and ascending aorta (58 mm) with a dissection flap extending from just above the left main coronary artery (LMCA) ostium and involving the proximal segment of right coronary artery (RCA) to the proximal aortic arch (Fig. 2A & B). Coronal and Sagittal Multiplanar Reconstruction (MPR) of the 75% phase of the gated CT Aortogram study showing type A aortic dissection extending to the aortic root (Fig. 3A & B). Axial Multiplanar Reconstruction (MPR) of the 45% phase of the gated CT Aortogram study showing the relationship of the intimal flap with the right coronary artery (RCA) and the left main coronary artery (LMCA) (Fig. 4A & B).

**Fig. 5:**
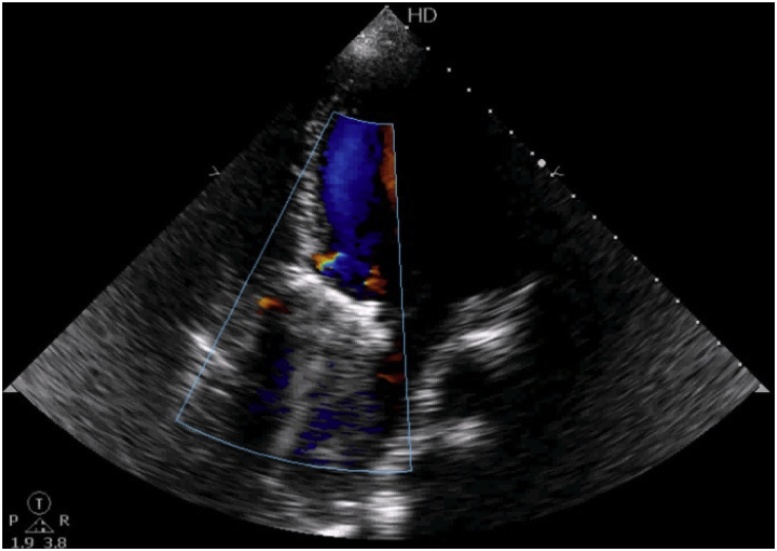
Post procedure echo shows normally functioning aortic disc prosthesis, with mild valvular AR & LV dysfunction (EF 40%).

After consulting with the cardiothoracic team, the patient was planned for Aortic root reconstruction (Bentall’s procedure) with CABG. The procedure was done using a St JUDE composite graft, a mechanical prosthetic valve and a saphenous venous graft (to the mid-RCA). The patient underwent the procedure successfully and had an uneventful recovery. Post-procedure echo showed normally functioning aortic disc prosthesis, with mild valvular AR with LVEF 40% (Fig. 5).

## 3 Discussion

In recent times, the incidence of Aortic dissection appears to be increasing. It has an incidence rate of 3–4 cases per 100,000 persons per year. It has a high mortality rate. It is common in elderly males. Aortic dilatation definitely increases risk but is not a must in every case. Most ascending aortic dissections occur when the aortic diameter is <5.5 cm [2]. Clinical manifestation of Aortic dissection can be variable, Therefore its diagnosis is challenging. 25% of cases, may have associated ECG changes suggestive of acute coronary syndrome leading to a possible misdiagnosis especially if associated ST elevation in ECG [3]. In 1 to 5% cases there may be associated myocardial ischemia [4]. Primary suspicion of Aortic dissection is usually based on a patient’s history which varies from typical complaints like a sharp, severe tearing kind of backache with chest pain to asymptomatic until associated with valvular regurgitation as in our patient [5]. Acute ascending aortic dissections are surgical emergencies. Descending aorta dissections are treated medically unless it is progressive or with hemorrhage into the pleural or retroperitoneal space. Thoracic aneurysms usually expand with increasing wall stress and eventual rupture [6,7]. Morphological classification of Aortic aneurysms is into fusiform or saccular categories. Anatomically the aortic root, ascending aorta, aortic arch or the descending aorta can be involved. Two commonly used classifications are Stanford and De Bakey. Stanford type A involves the ascending thoracic aorta, whereas type B dissections do not involve the ascending thoracic aorta. De Bakey type 1 dissections involve the whole aorta, type 2 dissections involve the ascending aorta and type 3 dissection involves the descending aorta. Thus, Stanford type A dissection includes De Bakey types 1 and 2, and Stanford type B equals De Bakey type 3. Shiga et al. [3] reviewed published studies of diagnostic methods of aortic dissection (i.e, TEE, helical CT and MRI)in a meta-analysis and showed that these tests have equal and reliable diagnostic value. TEE had 99% sensitivity and 95% specificity, helical CT had 100% sensitivity and 98% specificity, and MRI had 98% sensitivity and 98% specificity. Classification of aortic dissection is very important because treatment and prognosis depend on it. For example, classic type A dissection has to manage with immediate surgical intervention, whereas classic type B dissection can be managed medically. Localizing the entry point (tear), if possible, is very helpful because the ultimate aim of the intervention is to occlude the entry point. 2-D echo can be useful to visualize intimal flap, point of entry, and true and false lumens [8]. Stanford A dissection extending to cause acute myocardial infarction is seen only in 3% cases but can be life-threatening [9]. Most patients with concurrent infarction involve RCA. Differentiating the two (acute MI, Stanford A dissection) can be very difficult for emergency physicians and can lead to misdiagnosis in patients with ST-segment elevation [10]. Consequent treatment with antiplatelet, antithrombin and thrombolytic agents can cause life-threatening to bleed. Apart from good blood pressure control, prevention of aortic dissection can be done by elective aortic surgery in patients with dilated ascending aorta. Ascending TAA to prevent rupture or dissection is indicated when the ascending aortic diameter reaches 5.5 cm for non-Marfan patients and 4.5 cm in Marfan patients [11]. Spontaneous coronary artery dissection can be seen in cases of aortic aneurysm and aortic regurgitation [12]. Aorto arteritis is a non-atherosclerotic chronic inflammatory vascular disease of unknown etiology that affects the aorta, proximal parts of its major branches. In this case, there is a possibility that there was underlying spontaneous coronary artery dissection which in turn could be cause for silent ischemia in young women.

## 4 Conclusion

Acute aortic dissection is a catastrophic disease with very high morbidity and mortality needing a high index of suspicion for its diagnosis. No specific clinical finding has high sensitivity and therefore clinician evaluation of the patient remains foremost in its successful diagnosis and management.

## Funding

None.

## Ethical approval

Ethical Commitee approved.

## Consent

Consent obtained.

## Author’s contribution

Dr. Babu Reddy: Data Collection.

Dr. H.S Natraj Setty: Writing the paper.

Dr. B.C Srinivas: Data analysis.

Dr. T.R Raghu: Study Design.

Dr. Veeresh Patil: Data analysis.

Dr. Sandeep Shankar: Data analysis.

Dr. Vijay Kumar: Reference Collection.

Dr. C.M Nagesh: Reference Collection.

Dr. C.N Manjunath: Final Approval.

## Registration of research studies

N/A.

## Guarantor

Dr. Natraj Setty H.S.

## Provenance and peer review

Not commissioned, externally peer-reviewed.

## Declaration of Competing Interest

None.
